# The Effect of “Countrywide Services Management Law” on the Work Motivation of the Employees of Iranian Ministry of Health

**DOI:** 10.5812/ircmj.13983

**Published:** 2014-02-05

**Authors:** Hossein Shafeghat, Mehdi Jafari, Abbas Monavarian, Maryam Shafayi, Reza Dehnavieh

**Affiliations:** 1 Health Management and Economics Research Center, Iran University of Medical Sciences, Tehran, IR Iran; 2 Department of Management, School of Management, University of Tehran, Tehran, IR Iran; 3 Department of Health Services' Management, School of Health Management and Information Sciences, Iran University of Medical Sciences, Tehran, IR Iran; 4 Research Center for Health Services Management, Institute for Future Studies in Health, Kerman University of Medical Sciences, Kerman, IR Iran

**Keywords:** Health, Work, Motivation, Iran

## Abstract

**Background:**

Labor laws and regulations have inevitable effects on employees’ work motivation as well as the overall efficiency and productivity of the organization.

**Objectives:**

This study was conducted to assess the effects of the “Countrywide Services Management Law” on the work motivation level of the employees of the Iranian Ministry of Health.

**Patients and Methods:**

This cross-sectional study was done in 2011 in the Iran's Ministry of Health. Data was collected by a 51-item Likert scale questionnaire, in five domains including: organizational structure, information technology, training patterns, salary and bonus system and re-engineering process. The reliability and validity of the questionnaire was evaluated (Cronbach's alpha= 0.96). Data analysis was conducted using descriptive and inferential statistics (t-test).

**Results:**

Out of 192 samples examined, 55.2% of the respondents were female, 88 (45.8%) had BS degree and 116 (60.4%) had less than 10 years’ experience. The mean scores in the domains of organizational structure, information technology, training patterns, salary and bonus system and re-engineering patterns were: 3.11, 3.51, 3.05, 3.21 and 3.14, respectively. Relationship between the items related to manpower in the “Countrywide Services Management Law”, with employees' work motivation was significant (P < 0.0001). The training patterns did not show a significant relation (P < 0.26) with any of five domains.

**Conclusions:**

According to our results and the views of the employees of the Iranian Ministry of Health, “Countrywide Services Management Law” positively affected the personnel's work motivation regarding all the factors associated with motivation including: organizational structure, information technology, training patterns, salary and bonus system and re-engineering pattern. Finally, to enhance the workforce motivation and satisfaction level, application and implementation of the rules and regulations should be based on the organizational needs.

## 1. Background

Lack of motivation in employees of the health environments leads to work absenteeism and delay in and inadequacy of the care provided to patients (1, 2). On the other hand, to provide adequate care to patients in the hospital environments, employees should have high level of work motivation (3). To reach the organizational goals such as profitability, greater employee productivity, efficiency and performance, managers should always pay attention to the work motivation of the employees (4-6). The first labor law in Iran was drafted back in 1922. Due to failures to meet the needs of the employees and work unions and in order to solve the administrative and employment problems, Iranian “Countrywide Services Management Law” was drafted which includes:15 chapters, 128 articles and 106 amendments. It was passed in 2007 by a joint commission to address the civil services management bill in the parliament based on the 85 principles. The law was finally passed by the Guardian Council on 2007/18/07 and was issued to the government on 2007/07/25 through a presidential command (7). According to this law, staff employment and recruiting should be based on the equal opportunities solely based on the relative efficiency and professional qualifications and with consideration of the organization's productivity and interest (8, 9).

Previous researchers have evaluated the effects of various motivation-related factors on the labor force work productivity. For example, Poorsadeghi found that work motivation, education and work environment are effective factors in increasing the work efficiency and productivity of the organizations (10). Ala al-Maliki has found that financial, motivational and organizational structure of the organization, and education level and job related skills are the effective factors contributing to the employees' productivity. Organizational structure refers to the communication and interaction patterns between the employees of the various sectors of an organization (11, 12). Importance of the organizational structure as a factor contributing to the employees' work motivation is highlighted in another study and several other studies (13, 14). Personal motivational factors such as the employee's rights, benefits, and bonus system are known as the most important work related motivational factors and their importance is shown in various studies. In this regard, the pay level is considered as one of the most important factors that affect the work motivation of the staff and managers of the teaching hospitals (15). Regarding the education, each employee needs some work related skills and needs to be trained for them (16). Employees' training system is an integral part of the civil services law to enhance the work motivation of the employees (5, 17). In a research study titled "Survey of the impact of the training in information technology on the improving the performance level of the staff", information technology training was regarded as an effective factor in enhancing the motivation level of the employees and in predicting their professional success (18). Several research studies have been conducted to identify the relation of information technology training and work motivation (19). Re-engineering is a process through which the organizational procedures are analyzed to identify the best method for implementing a set of specific work related changes (20, 21). Increasing employees' job satisfaction through teamwork, giving employees more authority and enriching tasks and removing the work barriers are some of the advantages of the re-engineering process (22, 23). Re-engineering the organizational tasks has in Iran has been performed by the administrative councils and councils for the management development and workforce development, to bring about fundamental changes in the administrative system of our country (5).

## 2. Objectives

As mentioned above, identifying the factors affecting workforce motivation and implementing laws and regulation in this regard, can help in enhancing the staff satisfaction and performance level. Therefore, this study aimed at identifying the work-force related aspects of the Iranian “Country-wide Services Management Law” on the work motivation of the employees of the Iranian Ministry of Health.

## 3. Patients and Methods

This cross-sectional study was conducted in the deputy of development and resource management of the Iranian Ministry of Health in 2011. The data collection instrument was a 51-item questionnaire based on the Likert scale. The content pertaining to workforce in the Iranian “Countrywide Services Management Law” was used to develop a questionnaire by an expert panel consisting of managers, staff experts and the faculty affiliated with the Iranian Ministry of Health. The main axes of this questionnaire were the organizational structure, information technology, training system, payment mechanism and organizational re-engineering processes. The questionnaire structure is described in Table 1. 

**Table 1.** The Questionnaire Structure

“Country-wide Services Management Law”	Related Articles	Extracted Questions
**Organizational structure**	29-34	1-12
**Information technology**	37-40	13-20
**Education system**	58-63	21-27
**Payment mechanism**	64-83 and also 115	28-44
**Re-engineering**	64-83 and also 115	45-51

Finally, 51 questions related to the workforce were developed and respondents answered the questions and determined the impact of the studied factors on their work motivation (very high, high, medium, low and very low), which were then converted to the numerical values ranging from 1 to 5. The study population was the staff members of the Iranian Ministry of Health, consisting of 192 subjects recruited based on the Morgan's simple random sampling method. The reliability and validity of the questionnaire was evaluated and confirmed (Cronbach's alpha = 0.96). Data analysis was performed using descriptive and inferential statistical methods (t-test). 

## 4. Results

Totally, 192 employees participated in our study and summary of their characteristics is provided below:

Gender: 106 women (55%) and 86 men (45%); Marital status: 149 married (77.6%) and 43 single (22.4%); Age: under 30 = 37 participants (19.3%), 30-40 = 106 participants (55.2%) and above 40 = 49 participants (25.5%). Degree: diploma and under diploma 9 participants (4.7%); associate degree: 49 participants (25.5%); BSc: 88 participants (45.8%); MSc: 32 participants (16.7%); PhD: 14 participants (7.3%). Work experience: under 10 years = 116 participants (60.4%); 10-15 years = 31 (16.1%); 16-20 years = 29 (15.1); 21-30 years = 16 (8.3%). Organizational positions: regular employee: 63(32.8%); expert: 99 (51.6%) and manger: 30 (15.6%). Table 2 shows descriptive statistics for the study variables (items related to the human resources (total score), organizational structure, information technology, education, payment system, and re-engineering) and their effects on the work motivation of the employees. Based on the results listed in Table 2, for all six variables the current situation is better that the expected According to the results (Table 3), the total scores questions related to the “Country Services Management Law” are significantly correlated with the employees' work motivation (P = 0.000, t = 4.35). When separately analyzed for different domains, the effect of organizational structure domain (P = 0.044, t = 2.03), payment mechanism (P = 0.001, t = 3.54), information technology (P = 0.000, t = 10.60) and re-engineering (P = 0.018, t = 2.38) on the work motivation of employees was significant but there was no significant relation between the work-related training and work motivation (P = 0.263, t = 1.12).

**Table 2.** Status of Total Scores and Scores in Each Domain Related to the Workforce-related Content of the Iranian Law of Services Management

Variable	Mean ± SD	Median	Min	Max
**Items related to the work-force,total score**	3.20 ± 0.674	3.18	1.88	4.53
**Organizational structure**	3.11 ± 0.733	3.17	1.33	4.75
**Information Technology**	3.51 ± 0.667	3.62	1.75	4.88
**Education**	3.05 ± 0.625	3	1.29	4.43
**Payment system**	3.21 ± 0.837	3.24	1.29	4.76
**Re-engineering**	3.14 ± 0.833	3.29	1.14	5

**Table 3.** Result of the t-test Analysis of the Items Related to the Workforce in the Iranian Services Management Law

Variable	Mean ± SD	Mean ± SE	t value	Confidence Interval	P value
**Items related to the workforce, total score**	3.2 ± 0.674	0.2 ± 0.047	4.35	191	0.00
**Organizational structure**	3.11 ± 0.733	0.11 ± 0.053	2.03	191	0.044
**Information Technology**	3.51 ± 0.667	0.05 ± 0.045	1.12	191	0.263
**Training**	3.05 ± 0.625	0.21 ± 0.06	3.54	191	0.00
**Payment system**	3.21 ± 0.837	0.51 ± 0.048	10.60	191	0.00
**Re-engineering**	3.14 ± 0.833	0.14 ± 0.06	2.38	191	0.018

## 5. Discussion

One of the most important goals of passing laws similar to the Iranian countrywide services Management Law, is encouraging and motivating the workforce as well as improving the performance and productivity level. It is clear that satisfied, motivated and ambitious human workforce would take contribute to the organizational development and higher level performance. In this study the effect of “Countrywide Services Management Law” on employees' work motivation was investigated by examining one main hypothesis and five ancillary hypotheses. According to our results, the positive relation of the examined factors with the workforce motivation of the employees was approved. Miller Franco investigated the influence of the health system policies and reforms on employees' work motivation through changing the rules and procedures (23). So the legitimacy of passing this law could be approved be achieving one of the defined objectives, which leads to infusing more work motivation and enthusiasm among the employees. According to the results, we can conclude that the suggested organizational structure contained in the law can lead to increased employees’ work motivation (to above than the average). This goal can be achieved by creating a re-engineering committee for modifying the organizational structure on the basis of a process-based approach and making the work environment more friendly and productive. Jerico (24) surveyed the effect of the implemented nursing services structure on organizational culture, and suggested the employee work motivation as one of the organizations' cultural aspects which would be influenced by the organizational structure. In the study of Shafaee, the impact of organizational structure on employees' commitment was approved which is consistent with the results of our study (14). In a study of employees' work motivation in the for-profit and non-profit organizations, Leete suggested that the structure and type of organization have a great influence on the employee's motivation level, and showed a significant relationship between the type of organization and work motivation level of the employees (25). Organizational reform has been an inevitable part of the health system reforms during the past three decades (5).

According to our results, the training systems as defined by the “Countrywide Services Management Law” did not have any significant effect on the work motivation of the staff (P = 0.263). This is contrary to the findings of the Manabe’s (26) study showing that the training programs increase work motivation and knowledge of service providers in encountering and treating patients. Lack of consistent, practical and effective educational programs can be regarded as the main reason of these results. On the other hand, the employees might not take the training programs seriously and the great majority of them believe that the existing training programs are redundant and they attend these programs mainly to obtain the certificates for their professional development; not for the purpose of becoming more skillful and knowledgeable. The Iranian Ministry of Health has to emphasize its training programs from the onset of employing the staff and also during their career through a systematic and strategic approach. As mentioned in the results, the recommended payment mechanism in the law has a significant role in the enhancement of the work motivation of the staff. According to the study of Chandler, financial status has been introduced as a prerequisite for the employees' productivity (27). So, the financial bonus system plays an important role in the staff's work motivation, fulfilling their physiological and also safety needs as stated in the Maslow's hierarchy of basic human needs. The information technology development mentioned in the “Countrywide Services Management Law” has a significant effect on the staff's work motivation. The reason might be the fact that being skillful in using information technology measures can enhance the performance level of the employees. According to the study of Mitchell (28), there is a significant relation between the internal and external work motivation level of the staff and use of information technology (29). Re-engineering process, which has been originated from the law, has also had a significant role in enhancing the staff's work motivation. According to the Reis study, re-engineering methods are remarkable measures in increasing employees' motivation for executing tasks and maximizing the productivity (29-32). Re-engineering, through more accurate use of the resources and correcting the working processes, enhances the interest and work motivation of employees in the organizations (33, 34). Also, employees’ work motivation has an important role in the successful implementation of various programs in the organization. The Iranian Countrywide Services Management Law has a significant role in increasing the staff motivation as well as enhancing the organizational dynamism. The prospective approach of this law and its positive role in motivating the employees can be attributed to its comprehensiveness and its emphasis on the most important organizational factors such as the structure, payment mechanism, re-engineering and information technology. These findings show and highlight the power of rule-setting in enhancing the workforce of the organization. The policy makers and legislators have to pay more attention to the effects of their rules and policies and try to utilize an evidence based approaches.
